# Antitumor and Radiosensitization Effects of a CXCR2 Inhibitor in Nasopharyngeal Carcinoma

**DOI:** 10.3389/fcell.2021.689613

**Published:** 2021-05-26

**Authors:** Xiaobei Liu, Tianxia Lan, Fei Mo, Jingyun Yang, Yuquan Wei, Xiawei Wei

**Affiliations:** Laboratory of Aging Research and Cancer Drug Target, State Key Laboratory of Biotherapy and Cancer Center, National Clinical Research Center for Geriatrics, West China Hospital, Sichuan University, Chengdu, Sichuan, China

**Keywords:** nasopharyngeal carcinoma, CXCR2, radiosensitization, angiogenesis, neutrophils

## Abstract

CXCR2, a member of the G-protein-coupled cell surface chemokine receptor family, is commonly found on leukocytes, endothelial cells and tumor cells including nasopharyngeal carcinoma cells. However, how the activity of CXCR2 and its ligand CXCL8 affects the development of nasopharyngeal carcinoma (NPC) remains unknown. Here, we found that CXCR2 and CXCL8 were both predicted poor prognosis in NPC patients. Furthermore, we identified that treatment with CXCR2 antagonist SB225002 of nasopharyngeal carcinoma cell lines resulted tumorigenesis inhibition *in vitro* and *in vivo*. In addition, we found that SB225002 could enhance NPC cells radiosensitivity through regulating cell circle distribution and interfering with cellular DNA damage repair. SB225002 also exhibited an efficient radiosensitization effect in C666-1 and HONE-1 bearing mice. Functionally, we showed that SB225002 reduced microvessel density and proliferation and induced tumor apoptosis. Furthermore, changes in the tumor microenvironment were also observed in this study. We observed that SB225002 reduced tumor-associated neutrophils (TANs) in the tumors tissue which were recruited especially after irradiation. Taken together, our results suggested that targeting the CXCL8-CXCR2 pathway is a promising therapeutic strategy for comprehensive NPC treatment.

## Introduction

Nasopharyngeal carcinoma (NPC), originating from the nasopharyngeal epithelium, is the most common head and neck malignancy (Kamran et al., 2015; Chua et al., 2016). Epidemiologically, NPC is highly prevalent in Southern China and Southeast Asia, particularly in the Cantonese region where the peak annual incidence rate reaches 30 per 1,00,000 individuals (Lee A.W.M. et al., 2012; Bei et al., 2016). Radiotherapy (RT) with or without chemotherapy is the standard treatment for patients with nasopharyngeal carcinoma. Despite dramatic improvements in the clinical outcomes achieved by modern radiotherapy technology, more than 20-30% of NPC patients still have local recurrence and distant metastasis (Lee et al., 2015). However, due to toxicity, side effects and radioresistance, existing standard chemoradiation strategies have reached the upper limit (Akervall et al., 2014). Therefore, there is an urgent need to develop novel small molecule inhibitors that can enhance the therapeutic effects of radiation against NPC with minimal toxicity to further improve the prognosis for NPC patients.

C-X-C motif chemokine receptor 2 (CXCR2), a member of the G-protein-coupled cell surface chemokine receptor family, is commonly found on leukocytes, endothelial cells and tumor cells (Cacalano et al., 1994; Rot and von Andrian, 2004). C-X-C motif chemokine ligand 8 (CXCL8) is a pleiotropic cytokine with high affinity for its receptor CXCR2. Accumulating evidence suggests that the CXCL8-CXCR2 pathway is crucial for the initiation and development of tumors. For instance, CXCR2 and CXCL8 are overexpressed in various human cancers, such as oesophageal cancer, pancreatic cancer and ovarian cancer, and this overexpression positively correlates with aggressive tumor behavior and poor prognosis (Wente et al., 2006; Waugh and Wilson, 2008; Nishi et al., 2015; Ignacio et al., 2018). Additionally, the CXCL8-CXCR2 pathway is associated with angiogenesis and tumor-stromal interactions, where it promotes tumor progression and metastasis (Wang et al., 2006; Waugh and Wilson, 2008; Singh et al., 2009; Ijichi et al., 2011; Lee Y.S. et al., 2012; Steele et al., 2016). More importantly, genetic or pharmaceutical blockade of CXCR2 reduces tumorigenesis and angiogenesis in mice with oesophageal cancer, lung cancer, breast cancer and pancreatic cancer (Keane et al., 2004; Wislez et al., 2006; Matsuo et al., 2009; Xu et al., 2018). A few studies have suggested that the CXCL8-CXCR2 pathway may also play a role in NPC tumor progression. Yoshizaki et al. (2001) observed that CXCR2 and CXCL8 are abundantly expressed in clinical samples of NPC (Horikawa et al., 2005). Lo et al. (2013) demonstrated that CXCR2 and CXCL8 are expressed in NPC cell lines and induce the growth of tumour spheroids. The above studies indicate that the CXCL8-CXCR2 pathway is a promising target for anticancer treatment.

SB225002 is a small-molecule inhibitor with selective affinity for CXCR2 that competes with ligands, including CXCL8, stopping them from binding to CXCR2. SB225002 has been applied to inhibit oncogenesis and metastasis in ovarian cancer and oesophageal cancer (Wang et al., 2006; Yung et al., 2018). Moreover, SB225002 has been indicated to be a negative regulator of the MAPK signaling pathway, and it has been reported that MAPK signalng is involved in the radioresistance (Eder et al., 2017; Pei et al., 2017). Thus, we hypothesized that SB225002 may increase the radiation sensitivity of NPC cells. In this study, we aimed to confirm the expression of CXCR2 in human nasopharyngeal carcinoma and attempted to study the antitumor and radiosensitization effects and mechanisms of SB225002 in NPC.

## Materials and Methods

### Cell Lines and Cultures

Human NPC cell lines (C666-1, CNE-1, CNE-2, HNE-1, and HONE-1) were obtained from the American Type Culture Collection (ATCC). Cells were cultured in Dulbecco’s modified Eagle’s medium (DMEM, Gibco) supplemented with 10% foetal bovine serum (FBS; Gibco) and 1% antibiotics (penicillin and streptomycin). Human umbilical vein endothelial cells (HUVECs) were obtained from the State Key Laboratory of Biotherapy and were maintained in low-glucose DMEM. Cells were maintained at 37°C in a humidified atmosphere with 5% CO_2_.

### Prepare of SB225002

SB225002 was purchased from Selleck Chemicals. For *in vitro* studies, SB225002 was dissolved initially as a 10 mM stock solution in dimethyl sulfoxide (DMSO, Sigma-Aldrich), stored at −20°C and diluted in cell culture medium to achieve a final concentration. For *in vivo* studies, SB225002 was prepared in 25% (v/v) PEG 400 (Sigma-Aldrich) and 5% (v/v) Tween 80 (Sigma-Aldrich), containing 2% (v/v) DMSO.

### Cell Proliferation Assay

Cell viability was measured using the Cell Counting Kit-8 (CCK8, MedChemExpress) assay. Briefly, tumor cells (1–3 × 10^3^ cells/well) were seeded in 96-well plates and cultured overnight. Then, the cells were treated with various concentrations of SB225002 for 24, 48, or 72 h. DMSO (0.1%) served as the vehicle control corresponding to the concentration present in the highest SB225002 dosage of 1 mM to exclude possible effects of the solvent. Thereafter, 10 μL of CCK8 reagent was added to each well, and the plates were incubated in a 5% CO_2_ atmosphere at 37°C for an additional 0.5-2 h. The absorbance was measured at 450 nm using a Spectra MAX M5 microplate spectrophotometer (Molecular Devices). The percentage of the control samples of each cell line was calculated thereafter.

### Clonogenic Survival Assays

Tumor cells (400 cells/well) were seeded in 6-well plates, incubated for 24 h and treated with various concentrations of SB225002. After 10-12 days of incubation, the cells were fixed with ethanol and stained with 0.5% crystal violet. Colonies comprising more than 50 cells were manually counted by microscope. For the radiosensitivity assay, tumor cells (200-1000 cells/well) were seeded in 6-well plates. After culturing for 24 h, the cells were treated with SB225002 (0, 0.5, and 1 μM) for 3-4 h and then irradiated with a single dose of 0 (control), 2, 4, 6, or 8 Gy. Following irradiation, the cells were incubated with SB225002 for an additional 20 h, and then the medium was replaced by fresh medium. The following steps are the same as described above. After correcting for initial plating efficiency, the colony survival data were used to plot clonogenic survival curves.

### Cell Cycle Analysis

Cell cycle distribution was determined by flow cytometry with PI staining. Briefly, cells were treated with SB225002 at various concentrations or in combination with irradiation at a dose of 8 Gy. After culturing for 8 h, cells were fixed in 70% ice-cold ethanol at 4°C overnight. Then, the cells were stained with PI staining solution (Sigma Aldrich) containing RNase A for 30 min in the dark and analyzed by flow cytometry. Data were analyzed with Novo Express 1.1.2.

### Endothelial Cell Migration and Tube Formation Assays

HUVEC cell mobility and migration was evaluated using a modified Boyden chamber (8-μm pore size; Merck Millipore). A total of 1 × 10^5^ HUVECs in 200 μl of serum-free medium were added to the top chamber, and 600 μl of serum-free medium, tumor culture medium supernatant or 50 ng/ml recombinant human IL-8 (Novoprotein) was added to the bottom chamber. Both chambers contained 0.5 μM SB225002 or 0.1% DMSO. After 24 h of migration, filters were rinsed with PBS, fixed with 4% paraformaldehyde and stained with 0.5% crystal violet. The migrated cells on the underside of the whole transwell inserts were quantified by manual counting and photographed under a light microscope. The ability of HUVECs to form capillary-like endothelial tubes was determined *in vitro*. Briefly, a total of 1 × 10^5^ HUVECs were seeded on a Matrigel (Thermo Fisher Scientific) pre-coated 24-well plate and incubated in serum-free medium, tumor culture medium supernatant or 50 ng/ml recombinant human IL-8 with SB225002 (0.5 μM) or 0.1% DMSO for an additional 3-4 h. Photomicrographs from each well were captured and analyzed using ImageJ software, version 2.02 (National Institutes of Health, United States).

### Western Blot Assay

NPC cells treated with SB225005 at various concentrations for 2 h were washed twice in ice-cold PBS and lysed in RIPA lysis buffer (Invitrogen) containing 1 mM phenylmethanesulfonylfluoride (PMSF, Sigma-Aldrich) and a protease inhibitor cocktail (Sigma-Aldrich). Protein extracts were quantified by the bicinchoninic acid (BCA) protein assay kit (Thermo Fisher Scientific), separated on 12.5% SDS-PAGE and transferred to 0.2 μm polyvinylidene fluoride (PVDF) membranes (Merck Millipore). After blocking with 5% bovine serum albumin (BSA, Sigma-Aldrich) in TBS/T for 2 h at room temperature, the membranes were incubated with specific primary antibodies overnight at 4°C, followed by incubation with appropriate horseradish-peroxidase (HRP)-conjugated secondary antibodies for 2 h at room temperature. The reactive bands were detected by an enhanced chemiluminescence kit (Millipore). The primary antibodies for Erk1/2, p-Erk1/2 (Thr202/Tyr204), P38, p-P38 (Thr180/Tyr182), JNK, p-JNK (Thr183/Tyr185) and β-actin were purchased from Cell Signaling Technology.

### Immunofluorescence Staining

NPC cells (2 × 10^4^ cells/well) were seeded on coverslips in 24-well plates for cell adherence. Then, the cells treated with or without SB225002 (0.5 μM) for 3-4 h before irradiation were exposed to 8 Gy radiation. Cells were harvested at the indicated time points after irradiation, fixed with 4% paraformaldehyde for 15 min and permeabilized with 0.5% Triton X-100 for 10 min. After blocking with 5% BSA at 37°C for 1 h, cells were stained with a γ-H2AX antibody (1:500; ab11174, Abcam) overnight at 4°C. After being washed with PBS twice, cells were incubated with an anti-rabbit FITC secondary antibody at 37°C for 2 h. Nuclei were counterstained with DAPI (Beyotime Biotechnology) in room tempreture for 5 min, and images were visualized and captured by an LSM 710 laser-scanning confocal microscope (Carl Zeiss Microscopy).

### Mouse Models and Treatments

All animal experiments were approved by the Institutional Animal Care and Treatment Committee of Sichuan University, China. Nude mice (BALB/c, 4-5 weeks old) were purchased from Beijing Huafukang Bioscience and kept in a specific-pathogen-free (SPF) facility with consistent room temperature and humidity. A total of 5 × 10^6^ C666-1 or 1 × 10^7^ HONE-1 cells were injected subcutaneously into the right dorsal flank of BALB/c nude mice. After 5 days of tumor induction, the tumor-bearing mice were randomized into five groups (7 mice per group) and treated as follows: (Chua et al., 2016) blank, (Kamran et al., 2015) vehicle (25% PEG 400 and 5% Tween 80), (Lee A.W.M. et al., 2012) 10 mg/kg SB225002, (Bei et al., 2016) 8 Gy irradiation, and (Lee et al., 2015) a combination of 10 mg/kg SB225002 and 8 Gy irradiation. The administration of SB225002, by intraperitoneal injection once a day, began after the tumors reached approximately 0.6–0.7 cm in diameter. From the 3rd day of SB225002 administration, a single dose of 8 Gy radiation was administered. Tumor volumes were assessed by measurement of tumor diameters using a caliper every 3 days and calculated according to the formula: (long diameter) × (short diameter)^2^ × 0.52. Body weight was measured every 3 days. On the 23rd day, the mice were sacrificed after final administration. The tumors and organs were harvested, weighed, dissected and fixed in 4% paraformaldehyde for histochemistry; another part of the tumor was stored in liquid nitrogen for further use. The inhibition ratio was calculated according to the following formula: inhibition ratio (%) = [(A-B)/A] × 100, where A represents the average tumor volume of the vehicle control, and B represents that of the treated group.

### Tissue Microarray and Immunohistochemical (IHC) Staining

Commercially available tissue microarray slides (Outdo Biotech) of 99 NPC samples without adjacent normal nasopharyngeal tissues were obtained with detailed patient information, including age, gender, metastasis status, pathological pattern, tumor size, and TNM stage. The IHC analysis was carried out to assess the correlation between the protein expression of CXCR2 or CXCL8 and the prognosis of NPC patients based on the detailed survival data. In brief, the tissue sections were blocked with goat serum at 37°C for 40 min and then incubated overnight at 4°C with anti-human CXCR2 antibody (1:200; ab143935, Abcam) or anti-human CXCL8 antibody (1:500; ab18672, Abcam), followed by incubation with an HRP-conjugated secondary antibody. The tissue sections were then stained with a solution of 3,3-diaminobenzidine tetrahydrochloride and counterstained with haematoxylin. The expression of CXCR2 or CXCL8 was quantified based on the extent of staining (percentage of positive tumor cells) and the intensity of staining. The immunohistochemical score was independently assessed by 2 pathologists without knowledge of the patient characteristics. Additionally, the protein expression levels of Ki-67, CD31, VEGF, and Ly6G in xenograft mouse models were examined by IHC. The specific primary antibodies were anti-mouse Ki-67 antibody (1:200; ab16667, Abcam), anti-mouse CD31 antibody (1:50; ab28364, Abcam), anti-mouse VEGF antibody (1:500; ab46154, Abcam) and anti-mouse Ly6G antibody (1:800; GB11229, Servicebio).

### TUNEL Assay

To examine the apoptosis induction effect of SB225002 plus irradiation on tumor cells *in vivo*, paraffin sections of tumor tissue specimens were stained with terminal deoxynucleotidyl transferase (TdT)-mediated deoxyuridine triphosphate-biotin nick-end labeling (TUNEL) using a TUNEL kit (Promega) according to the manufacturer’s instructions. Images were visualized and captured by a DM 2500 fluorescence microscope (Leica Microsystems CMS GmbH).

### Flow Cytometry Analysis

Antibodies specific for mouse PerCP-cy5.5-CD45, FITC-CD11b, PE-Ly6C, BV510 or BV421-Ly6G, BV421-TGFβ, anti-IL-10 and anti-VEGF were purchased from Biolegend. Anti-human APC-CXCR2 antibodies and anti-rat FITC-secondary antibodies were purchased from BD Pharmingen. Anti-mouse CXCR2 antibodies were obtained from R&D Systems and LIVE/DEAD Fixable Blue Dead Cell Stain was obtained from Thermo Fisher Scientific.

To analyse the neutrophils and their functions in tumor tissues, tumors were minced into small pieces and dissociated using 1 mg/mL collagenase type IV (Sigma-Aldrich) in serum-free DMEM medium at 37°C for 1 h. After washing with PBS, cell suspensions were filtered with a 70-μm nylon cell strainer (BD Falcon) to remove clumps of cells and debris for subsequent flow cytometry. For cell surface staining, cells were stained with antibodies on ice for 30 min in the dark; for intracellular cytokine staining, cells were then fixed and permeabilized with paraformaldehyde and Triton-X100 and stained with intracellular antibodies overnight. Analyses were carried out on a NovoCyte flow cytometer (ACEA Biosciences) and data were analyzed using Novo Express 1.1.2.

### Statistical Analysis

Data were presented as the mean ± SD of at least three independent experiments. Data were statistically evaluated using a 2-tailed Student’s t test and a one-way analysis of variance (ANOVA) test. A Kaplan-Meier survival analysis (log-rank test) was used to illustrate the prognostic relevance of SB225002 in NPC patients. A P value < 0.05 was considered statistically significant. All statistical analyses were performed using GraphPad Prism 7.0 and the R Performance Analytics package.

## Results

### Elevated CXCR2 and CXCL8 Co-expression Levels in Nasopharyngeal Carcinoma Correlate With Poor Prognosis

**FIGURE 1:**
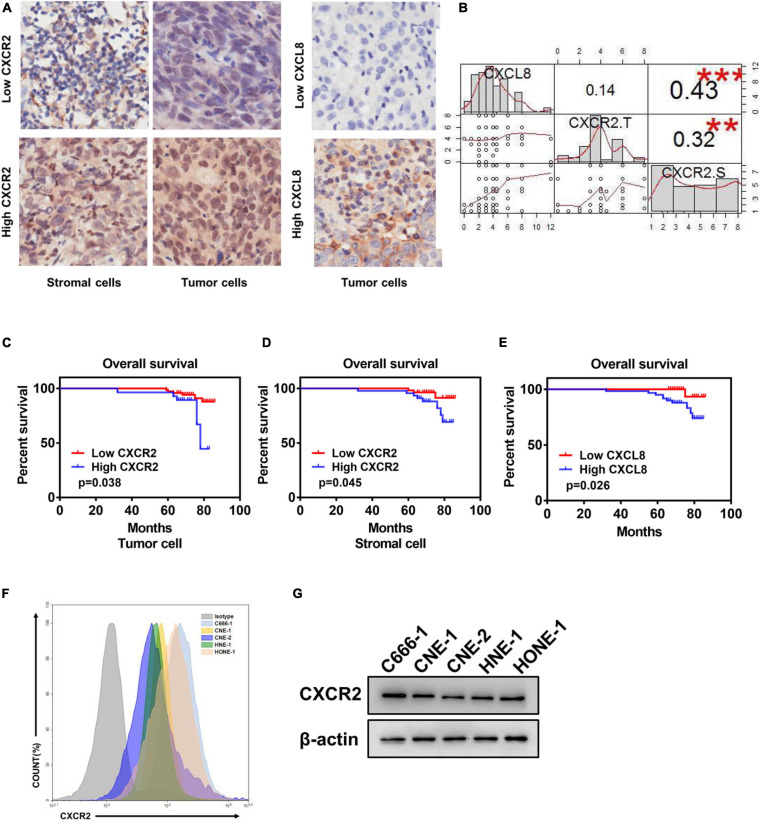
Elevated CXCR2 and CXCL8 co-expression levels in NPC correlate with poor prognosis. **(A)** Representative immunohistochemistry images showing CXCR2 expression in stromal and tumor cells in different individual NPC tissue (left) and CXCL8 expression in other NPC tissues (right). Magnification, 20×. **(B)** Correlation analysis between CXCR2 and CXCL8. CXCL8 expression and CXCR2 expression in tumor cells was significantly positively associated with CXCR2 expression in stromal cells, R = 0.43, *p* < 0.001 and R = 0.32, *p* < 0.01, but no association was found between CXCR2 expression in tumor cells and CXCL8 expression in stromal cells, R = 0.14, *p* > 0.05. Statistical analysis was performed using the Pearson’s correlation coefficient and linear regression. **(C–E)** According to IHC score, samples from NPC patients (*n* = 99) were divided into two groups based on low or high expression of the indicated markers, including CXCR2 in tumor and stromal cell and CXCL8. Overall survival was significantly negatively associated with the levels of CXCR2 in tumor cells, CXCR2 in stromal cells and CXCL8, as displayed in Kaplan–Meier survival curves of OS, **(C)** p = 0.038; **(D)** p = 0.045; and **(E)** p = 0.026. **(F–G)** CXCR2 expression analysis of six NPC cell lines using flow cytometry and western blot. (IHC score, immunohistochemical analysis score; R, Spearman’s correlation).

To explore the effect of CXCL8-CXCR2 signaling in nasopharyngeal carcinoma, we performed immunohistochemistry staining for CXCR2 and CXCL8 in an NPC tissue microarray and found CXCR2 localized in both tumor cells and stromal cells; CXCL8 was frequently observed in the cytosol of tumor cells (Figure 1A and Supplementary Table). Moreover, a significant positive correlation was detected between CXCR2 expression in stromal cells and CXCL8 expression in tumors in 99 NPC specimens (*P* < 0.05) but not between CXCR2 expression in tumor cells and CXCL8 expression in tumors (*P* > 0.05), which might indicate that tumor could regulate the behaviors of stromal cells in the tumor microenvironment via CXCL8-CXCR2 signaling (Figure 1B). Furthermore, the Kaplan-Meier analysis showed that the expression of CXCR2 and CXCL8 was significantly correlated with overall survival in NPC patients. As shown in Figures 1C-E, NPC patients with high CXCR2 expression levels in tumor cells or in stromal cells had shorter overall survival than patients with low CXCR2 levels (*P* < 0.05). Furthermore, NPC patients with high CXCL8 expression levels had shorter overall survival than patients with low CXCL8 levels (*P* < 0.05), suggesting that high CXCL8-CXCR2 signal levels correlate with poor prognosis. We then confirmed the expression of CXCR2 in nasopharyngeal carcinoma cell lines by flow cytometry (FCM) and Western blot (Figures 1F,G).

### CXCR2 Inhibitor Treatment Suppresses Tumorigenesis in Nasopharyngeal Carcinoma *in vitro* and *in vivo*

**FIGURE 2:**
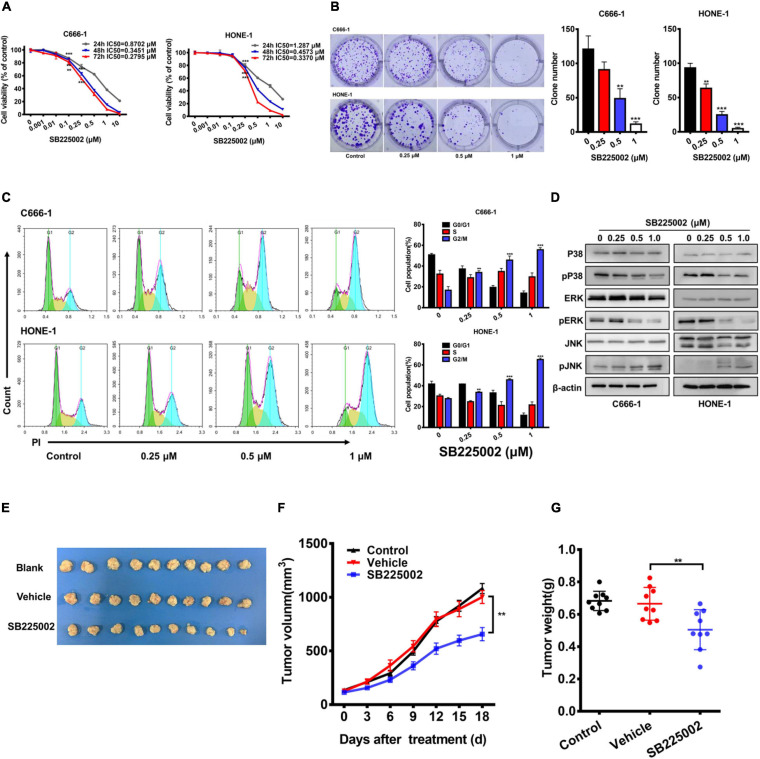
SB225002 suppresses tumorigenesis in NPC cells *in vitro* and *in vivo*. **(A)** SB225002 inhibited cell proliferation. C666-1 and HONE-1 human NPC cells were treated with SB225002 for (24, 48, and 72 h), cell viability was evaluated by CCK8 assay (**p < 0.01; ***p < 0.001; Student’s *t*-test). Values represent mean ± SD (*n* = 5). **(B)** The effects of SB225002 on colony formation in C666-1 and HONE-1 cells for 10-12 days. The colonies were counted and shown with histograms (**p < 0.01; ***p < 0.001; Student’s t-test). Values represent mean ± SD (n = 3). **(C)** Cell cycle analysis of C666-1 and HONE-1 cells after SB225002 treatment for 8 h (**p < 0.01; ***p < 0.001; Student’s *t*-test). Values represent mean ± SD (n = 3). **(D)** The expression of p38, p-p38, ERK, p-ERK, JNK and p-JNK in C666-1 and HONE-1 cells pre-treated with SB225002 for 2 h determined by western blot analyses and β-actin was employed as a standard. **(E–G)** C666-1 cells were established subcutaneous in female BALB/c mice and SB225002 at 10 mg/kg and vehicle administration once daily were started 5 days after inoculation. Tumor volumes and tumor weights were recorded every three days (seven mice per group). **(E)** Representative images of the xenograft tumors in three groups. **(F)** The growth curves of the tumors in groups were presented. Data are shown as the mean tumor volume ± SD (*n* = 7; **p < 0.01; two-way ANOVA). **(G)** The antitumor effects of SB225002 on the tumor weight in groups were shown as mean ± SD (*n* = 7; **p < 0.01; Student’s *t*-test).

To explore whether the CXCR2 inhibitor SB225002 has a direct effect on NPC cells, we treated a panel of 5 established NPC cell lines with various concentrations of SB2250002 for 24, 48, and 72 h and assayed cell viability by CCK8 assay. As shown in Figure 2A and Supplementary Figure 1, all these cell lines were sensitive to treatment with SB225002, and the cell proliferation was inhibited in a concentration- and time-dependent manner. In particular, the C666-1 cells and HONE-1 cells were most sensitive to SB225002, which corresponded with their CXCR2 expression levels. Thus, we chose C666-1 and HONE-1 cells for further study. Treatment of NPC cells with various concentrations of SB225002 also decreased the colony formation ability of NPC cells in a dose-dependent manner (Figure 2B). We also observed that SB225002 induced a sustained accumulation of NPC cells in the G2/M phase in the cell cycle analysis (Figure 2C). As shown in Figure 2D, SB225002 suppressed cell growth and proliferation through the inhibition of mitogen-activated protein kinase (MAPK) pathway activation in NPC cells. To further investigate the activity of SB225002 *in vivo*, C666-1-bearing mice were treated with 10 mg/kg SB225002. As shown in Figures 2E-G, both tumor size and tumor weight were substantially decreased in mice treated with SB225002 compared with those in mice treated with vehicle. Taken together, these results strongly indicate that SB225002 suppresses tumorigenesis in NPC *in vitro* and *in vivo*.

### CXCR2 Inhibitor Treatment Sensitizes NPC Cells to Radiation Through the Suppression of DSB Repair *in vitro*

**FIGURE 3:**
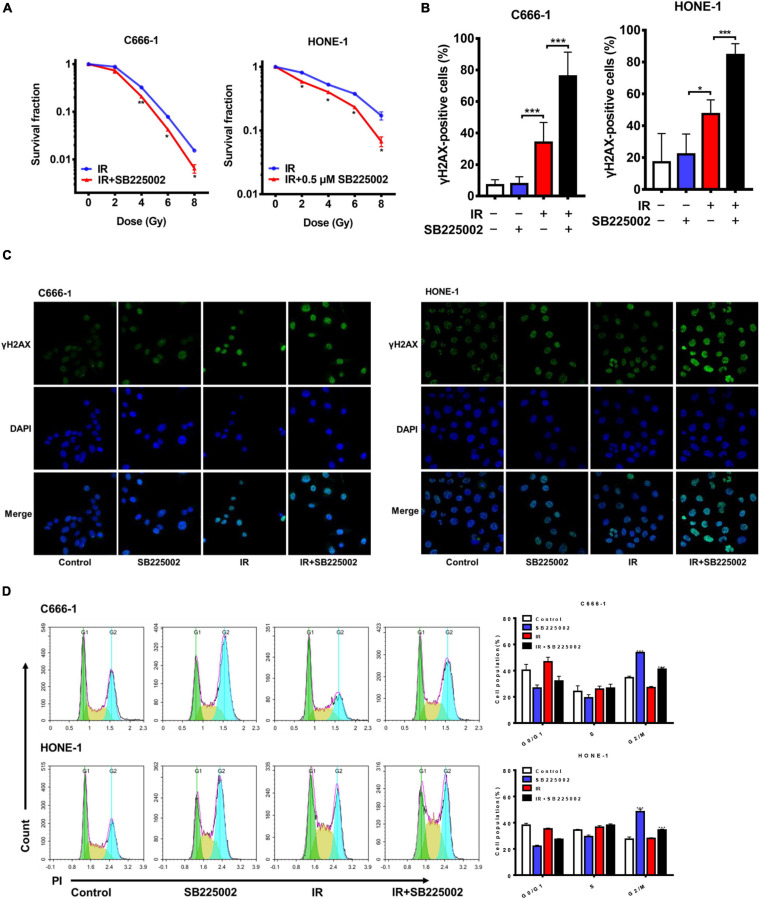
SB225002 sensitizes NPC cells to radiation *in vitro*. **(A)** SB225002 sensitized C666-1 and HONE-1 cells to radiation. Cells were treated with 0.5 μM SB225002 and irradiated with doses as indicated. The colonies were counted 12 days later. Values represent mean ± SD (*n* = 3; *p < 0.05; **p < 0.01; Student’s *t*-test). **(B,C)** DSB repair assay was performed by counting γ-H2AX foci. C666-1 and HONE-1 cells pre-treated with SB225002 for 3-4 h were irradiated with 8 Gy and harvested to immunofluorescence stain with DAPI and FITC for γ-H2AX at 1 h later. **(C)** Representative immunostaining images were shown, 20 × oil. **(B)** Cells with more than 10 foci were scored as positive and plotted with histograms. Data are shown with the mean ± SD of *n* = 5-8 fields obtained from three parallel experiments (*p < 0.05; ***p < 0.001; Student’s t-test). **(D)** Cell cycle distribution analyzed by flow cytometry of PI staining. C666-1 and HONE-1 cells pre-treated with SB225002 for 3-4 h were irradiated with 8 Gy and harvested to analyze by flow cytometry 4 h later (*n* = 3; ***p < 0.001; Student’s *t*-test).

MAPK signaling activity has been reported to be involved in radioresistance (Eder et al., 2017; Pei et al., 2017). Since SB225002 has been indicated to be a negative regulator of the MAPK signaling pathway, we speculated that SB225002 may increase the radiation sensitivity of NPC cells. To investigate whether SB225002 directly regulates NPC cell radiosensitivity, we performed a clonogenic survival assay to assess the survival of C666-1 and HONE-1 cells exposed to radiation at graded doses (0, 2, 4, 6, and 8 Gy). As expected, SB225002 treatment sensitized the NPC cells to the effects of radiation *in vitro* (Figure 3A). To further evaluate how SB225002 influence the cellular DNA damage repair, we investigated the levels of DNA double-strand breaks (DSBs) via immunofluorescence staining of γ-H2AX foci in NPC cells treated with SB225002 after exposure to X-rays. As shown in Figures 3B,C, compared to radiation alone, the combination of SB225002 treatment with irradiation (8 Gy) increased the expression of γ-H2AX nuclear foci at 1 h. Consistent with this result, the cell cycle analysis showed that SB225002 increased the sustained accumulation of cells in G2/M phase, which is a well-known occurrence in cells that are the most sensitive to radiotherapy. Notably, the treatment of irradiation significantly reduced the SB225002-induced accumulation of cells in the G2/M phase (Figure 3D). Collectively, these data suggest that SB225002 may interfere with cellular DNA damage repair, enhance radiation-induced cell killing and eventually contribute to the radiosensitization effect in NPC cells.

### CXCR2 Inhibitor Treatment Sensitizes NPC Tumors to Radiation *in vivo*

**FIGURE 4:**
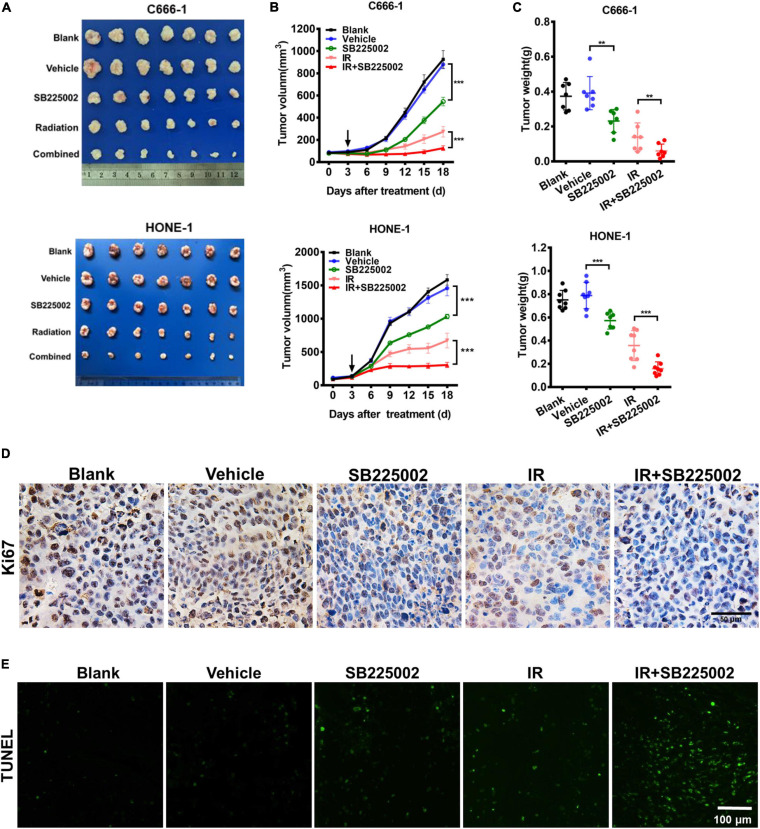
SB225002 sensitizes tumor to radiation *in vivo*. C666-1 and HONE-1 cells were established subcutaneous in female BALB/c mice and SB225002 at 10 mg/kg and vehicle administration once daily were started 5 days after inoculation. A single dose of 8 Gy was administered at the 3th day of SB225002 administration. Tumor volumes and tumor weights were recorded every three days (seven mice per group). **(A)** Representative images of the xenograft tumors in five groups. Mice bearing C666-1 (Upper) and HONE-1-1 (Blow) tumors. **(B)** The growth curves of the tumors in five groups were presented. Data are shown as the mean tumor volume ± SD (*n* = 7; ***p < 0.001; two-way ANOVA). **(C)** The antitumor and radiosensitization effects of SB225002 on the tumor weight in five groups were shown as mean ± SD (*n* = 7; **p < 0.01; ***p < 0.001; Student’s *t*-test). **(D)** Tumor cell proliferation was assessed by Ki67 immunohistochemical staining of paraffin-embedded C666-1 tumor sections (*n* = 3; Scale bar, 50 μm). **(E)** Tumor cell apoptosis was measured on paraffin-embedded C666-1 tumor sections by TUNEL staining (*n* = 3; Scale bar, 100 μm).

To assess the antitumor and radiosensitization effects of SB225002, we used subcutaneous xenograft null mice models of nasopharyngeal carcinoma (C666-1 and HONE-1). We noticed that SB225002 treatment alone caused a modest but significant reduction in tumor volume and tumor weight in both NPC mouse models, with tumor inhibition rates of approximately 38 and 30% in the C666-1 and HONE-1 models, respectively. Moreover, the combination of SB225002 plus irradiation led to an approximately 55% more reduction in both tumor volume and tumor weight when compared with that of irradiation alone (Figures 4A-C). Ki67 immunohistochemistry staining and the TUNEL assay were conducted on tumor tissues to obtain additional insight into proliferation and apoptosis *in vivo*. The images clearly showed that compared with the single treatment, the combination treatment resulted in a significant reduction in proliferating cells with positive nuclear Ki-67 staining in the C666-1 tumor model (Figure 4D). Furthermore, TUNEL-positive cells were significantly increased in the cells in the combination group (Figure 4E). In addition, to examine the potential toxicity of the combination of irradiation and SB225002, histological examinations of vital organs by H&E staining and serum biochemical detection were performed. As shown in Supplementary Figures 2A-C, no body weight decrease, histological abnormalities or hepatorenal function changes were found, suggesting that the combined treatment was well tolerated. Taken together, these results show that SB225002 increases tumor radiosensitivity and is associated with proliferation inhibition and apoptosis induction.

### CXCR2 Inhibitor Treatment Impairs the Recruitment of TANs Induced by Irradiation *in vivo*

**FIGURE 5:**
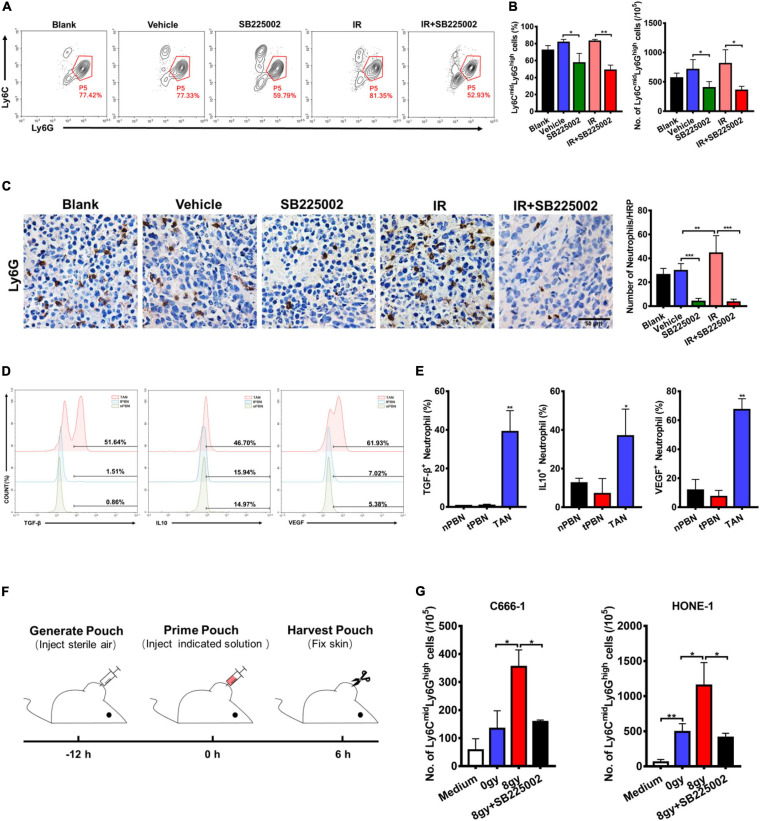
Effects of SB225002 on recruitment of TANs *in vivo*. **(A)** SB225002 significantly reduced TANs in C666-1 tumor-bearing nude mice. Total numbers of 10,000 cells gated on CD45-positive cells were collected and CD45^+^CD11b^+^Ly6C^*mid*^Ly6G^*high*^ cells were gated as TANs. Numbers illustrated indicate the percentage of the cells. **(B)** Flow cytometry analysis quantified TANs in tumor at the end of treatment with SB225002 combined radiotherapy. The percentage of TANs and the absolute number of TANs were shown as mean ± SD (*n* = 4; *p < 0.05; **p < 0.01; Student’s *t*-test). **(C)** TAN was assessed on paraffin-embedded C666-1 tumor sections by Ly6G immunohistochemical staining after SB225002 combined radiotherapy treatment. Representative immunohistochemistry images showing Ly6G-positive TANs in the xenograft tumors in five groups. Scale bar, 50 mm. Image analysis data for Ly6G-positive TANs were shown as mean ± SD (*n* = 3 animals each group, **p < 0.01; ***p < 0.001; Student’s *t*-test). **(D,E)** Intracellular flow cytometry detected the increased secretion of TGF-β, IL-10 and VEGF by Ly6G-positive neutrophils in C666-1 tumors (TAN) compared with the neutrophils in peripheral blood from both non-tumor bearing mice (nPBN) and C666-1 tumor-bearing mice (tPBN). Total numbers of 10,000 cells gated on CD45-positive cells were collected and the CD45^+^CD11b^+^Ly6C^*mid*^Ly6G^*high*^ cells in tumor were gated. Numbers illustrated indicate the percentage of the cells (*n* = 3; *p < 0.05; **p < 0.01; Student’s t-test). **(F)** Schematic representation of the experiment sequence in air pouch experiments of recruitment of neutrophils stimulated by supernatants of irradiated tumor cells *in vivo*. **(G)** Flow cytometry analysis quantified neutrophils recruitment into the air pouch skin samples. Air pouch skin samples were collected at the indicated time point, and neutrophils were analyzed by flow cytometry. Total cell numbers per 10^5^ cells are shown. Means ± SD are depicted (*n* = 3; *p < 0.05; Student’s *t*-test).

In the tumor microenvironment, infiltrating myeloid cells, such as neutrophils and macrophages, contribute to tumor progression. In particular, CXCR2 is expressed in neutrophils, and tumor-associated neutrophils (TANs) have been shown to have antitumorigenic (N1) or pro-tumorigenic functions during tumor development. Thus, we evaluated the effect of SB225002 on TANs, and the functions of TANs in NPC to investigate whether the host immune system contributes to the antitumor and radiosensitization effects of CXCR2 inhibition. Flow cytometry and immunohistochemical staining of tumor tissue was performed to assess TANs in C666-1 tumor-bearing mice after 18 days of combined treatment. As shown in Figure 5A, the number of TANs (CD45^+^CD11b^+^Ly6C^*mid*^Ly6G^*high*^) significantly decreased in both SB225002 alone and combined treatment groups when compared with the vehicle and radiation alone groups. Immunostaining of Ly6G in tumor tissues also suggested a decrease in TAN infiltration after SB225002 treatment, while irradiation treatment induced TAN accumulation (Figure 5B). Furthermore, intracellular flow cytometry was conducted to determine the functions of TANs in NPC tumors. We found that the expression level of pro-tumor cytokines including TGF-β, IL-10 and VEGF were significantly higher in TANs comparing to those in neutrophils in the peripheral blood of both non-tumor-bearing mice and C666-1 tumor-bearing mice, which confirmed that TANs in NPC tumors have pro-tumorigenic properties (Figures 5C,D).

In order to verify our hypothesis that SB225002 impairs the sequential recruitment of TANs induced by irradiation *in vivo*, we utilized an air pouch model in which supernatants of irradiated NPC cells with or without SB225002 were injected (Krombach et al., 2019), and neutrophil recruitment was analyzed in the air pouch skin (Figure 5E). As shown in Supplementary Figure 3, culture supernatants of irradiated tumor cells were harvested 4 days after irradiation with 0 Gy and 8 Gy, while serum-free culture medium served as a control. The number of recruited neutrophils increased significantly when supernatants of 8 Gy-irradiated cells were injected compared to when supernatants of 0 Gy-irradiated cells were injected (Figure 5F). However, the supernatants of 8 Gy-irradiated cells recruited fewer neutrophils when 0.5 μM SB225002 was added (Figure 5F). In combination, these data suggest that SB225002 may contribute to the suppression of radioresistance by regulating the recruitment of TANs stimulated by radiation *in vivo*.

### Regulation of Tumor Angiogenesis by CXCR2 Inhibitor Treatment

**FIGURE 6:**
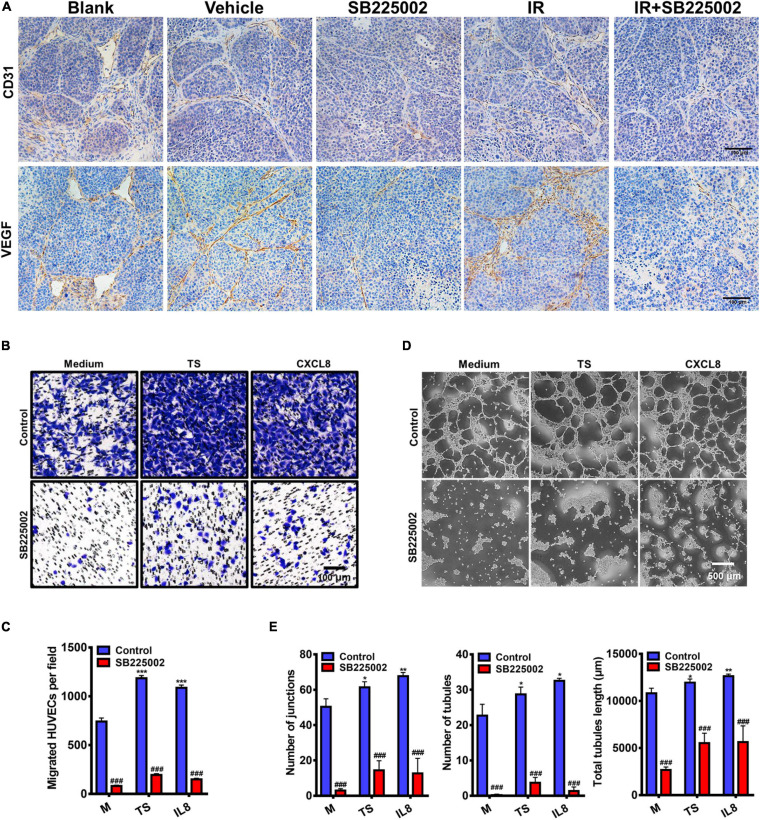
Regulation of tumor angiogenesis by CXCR2 inhibitor. **(A)** Paraffin-embedded C666-1 tumor sections after SB225002 combined radiotherapy treatment were examined by immunohistochemical analysis with anti-CD31 and anti-VEGF antibody. Representative tumor vasculature from five groups was shown. **(B)** Representative photographs of HUVECs migration *in vitro*. HUVECs were seeded into the upper chamber of a transwell system, and tumor culture medium supernatant (TS) of nasopharyngeal carcinoma cell C666-1, human recombinant protein CXCL8 or SB225002 (0.5 μM), was added to the bottom chamber. Scale bar = 100 μm. **(C)** Statistical analysis of the number of migrated HUVECs. Data were assessed by ImageJ, showing as Mean ± SD (*n* = 3; ***p < 0.001; ###*p* < 0.001. * represent p value between tumor supernatant or CXCL8 group and serum free medium group, # represent p value between groups with or without SB225002. **(D)** Representative photographs of HUVEC tubule formation *in vitro* grown on the Matrigel after 3-4 h in the presence of tumor supernatant, human recombinant protein CXCL8 (50ng/ml) or SB225002(0.5 μM), compared to free serum medium. Scale bar = 500 μm. **(E)** Statistical analysis of junctions, tubules and total tubule length formed by HUVECs. Data were assessed by ImageJ showing as Mean ± SD (*n* = 3; *p < 0.05; **p < 0.01; ###*p* < 0.001. * represent *p* value between tumor supernatant or CXCL8 group and serum free medium group, # represent p value between groups with or without SB225002.

To further investigate the mechanisms through which SB225002 inhibits NPC tumour growth and increases radiosensitivity *in vivo*, we selected two well defined markers of angiogenesis, CD31 and VEGF, to evaluate angiogenesis in the tumor sections. As shown in Figure 6A, the densities of CD31-positive and VEGF-positive microvessels in tumor sections from vehicle and radiation alone groups were both significantly higher than those in the SB225002 alone and combined treatment groups. Moreover, to validate the antiangiogenic capacity of SB225002, we first measured the migration of HUVECs in a modified Boyden chamber. The culture supernatants of C666-1 cells or the medium containing the chemokine CXCL8 was added to the lower chamber, and HUVECs were added to the upper chamber. After 24 h of incubation, the number of migrated HUVECs was significantly elevated following culture in tumor supernatants and medium containing CXCL8 relative to that of HUVECs cultured only in medium. In agreement with these observations, blockage of the binding between the CXCL8 receptor CXCR2 with SB225002 decreased the migration of HUVECs following exposure to tumor supernatants or CXCL8 (Figures 6B,C). We next examined how SB225002 influences the tubule formation of endothelial cells when plated on Matrigel. As observed in Figures 6D,E, SB225002-mediated inhibition of CXCL8 prevented tumor supernatant-induced formation of tubules. Taken together, these results clearly indicate that the development of the microvascular network is inhibited by SB225002 treatment *in vitro* and *in vivo*.

## Discussion

Although modern radiotherapy technology has achieved considerable improvements in NPC, many NPC patients still have local recurrence and distant metastasis (Gupta et al., 2002; Yeh et al., 2005; Lee A.W.M. et al., 2012), owing to the upper limit of toxicity with associated side effects and complications (Akervall et al., 2014). Therefore, efforts are continuing to develop new strategies to enhance the antitumor effect of radiation therapy on NPC.

CXCR2, binding to its ligands, is important in tumorigenesis and tumor progression (Luan et al., 1997; Maxwell et al., 2007; Cataisson et al., 2009). There is clear evidence of the role played by CXCR2 and its associated ligands not only in promoting tumor proliferation but also in modulating blood vessel formation and neutrophil recruitment to the site of the tumor (Horton et al., 2007; Raman et al., 2011). Blockade of CXCR2 signaling pathways is a promising target for antitumor therapy.

Several observations have been made in this study concerning SB225002, the selective CXCR2 inhibitor, used in the treatment of NPC. In this study, we reveal that SB220552 suppresses tumorigenesis and radioresistance in NPC. By immunohistochemical staining of tissue microarray slides of NPC samples, we first found that an increased level of CXCR2 and CXCL8 predicts poor clinical outcomes which implies that CXCR2 is an attractive therapeutic target for NPC. We next demonstrated by CCK8 staining, clonogenicity and cell cycle assays, that SB225002 inhibits the viability and proliferation of NPC cells. Moreover, our data indicate that SB225002 sensitizes NPC cells to radiation *in vitro* by regulating the G2/M checkpoint and DNA damage repair. In our established NPC tumor model in nude BALB/c mice, the therapeutic effect of radiation against NPC was enhanced by SB225002 treatment. Furthermore, reduced recruitment of TANs and suppression of angiogenesis was observed using flow cytometry and immunohistochemical staining *in vivo*. Therefore, we may find out the possibility that the antitumor and radiosensitization activity of SB225002 results from improved sensitivity to radiation, suppression of angiogenesis as well as the disruption of immunosuppression induced by TANs.

Classically, CXCR2 is expressed on leukocytes, endothelial cells and tumor cells (Murphy et al., 2005; Vandercappellen et al., 2008; Yang et al., 2017). A number of studies have shown that blocking CXCR2 genetically or pharmacologically can inhibit angiogenesis and tumor growth (Hertzer, 2013; Wagner et al., 2015). For example, significant inhibition of human melanoma tumor growth and lung metastasis with a decrease in melanoma cell proliferation and angiogenesis has been shown in Cxcr2^–/–^ mice compared to WT nude mice (Singh, 2014). Studies of colitis-associated tumorigenesis and pancreatic ductal adenocarcinoma show a clear role for CXCR2 in tumor growth associated with attenuated MDSC recruitment in Cxcr2^–/–^ mice (Ijichi et al., 2011; Katoh et al., 2013). In addition, inhibition of CXCR2 reduces tumorigenesis and angiogenesis in the lung, oesophageal and ovarian cancer (Keane et al., 2004; Wang et al., 2006; Yung et al., 2018). Moreover, combining a CSF1R inhibitor with a CXCR2 inhibitor was shown to block granulocyte infiltration of tumors triggered by the inhibition of CSF1R and significantly reduce tumor growth (Kumar et al., 2017). A recently published study showed that dying tumor cell-derived DAMP release induced by radiotherapy triggers endothelial cell activation and the recruitment of myeloid cells (Krombach et al., 2019). These findings may help to explain the inhibitory and radiosensitization effects of SB225002 targeting CXCR2 in this study.

In this study, we found that SB225002 increases sensitivity to radiation in NPC cells by increasing the sustained accumulation of cells in G2/M phase, which is a well-known radiotherapy effect that increases sensitivity and suppresses DSB repair *in vitro*. In addition, the combination of radiotherapy with SB225002 suppressed angiogenesis and the recruitment of TANs triggered by irradiation *in vitro* and *in vivo* and showed strong antitumor effects. Moreover, in our study, the examinations of vital organs showed that there were no histological abnormalities or hepatorenal function changes, suggesting that the combined treatment was well tolerated.

Taken together, our results provide insights into the role of SB225002 used for NPC therapy both as an antitumor agent and radiosensitizer. SB225002 treatment dramatically enhanced the effect of radiotherapy with no toxicity to vital organs in treated mice. To the best of our knowledge, this is the first study to provide new perspectives on a CXCR2 inhibitor in combination with radiotherapy. Thus, we conclude that CXCR2 inhibition could be a potential therapeutic approach that might be further used in comprehensive NPC treatment.

## Data Availability Statement

The raw data supporting the conclusions of this article will be made available by the authors, without undue reservation.

## Ethics Statement

The animal study was reviewed and approved by Ethics Committee of Sichuan Unicersity.

## Author Contributions

XW and YW have designed the study and revised the manuscript. XL, TL, and FM have conducted all the experiments. XL concluded the data and wrote the manuscript. TL and XW have helped to revised it. All authors contributed to the article and approved the submitted version.

## Conflict of Interest

The authors declare that the research was conducted in the absence of any commercial or financial relationships that could be construed as a potential conflict of interest.
