# Supplementary material for: Antitumor and Radiosensitization Effects of a CXCR2 Inhibitor in Nasopharyngeal Carcinoma
Source: Front Cell Dev Biol. 2021 May 26;9:689613. doi: 10.3389/fcell.2021.689613 (PMC8188356; doi:10.3389/fcell.2021.689613)
Supplement: Supplementary file 1 [file Image_1.pdf]

Supplementary Figure 1

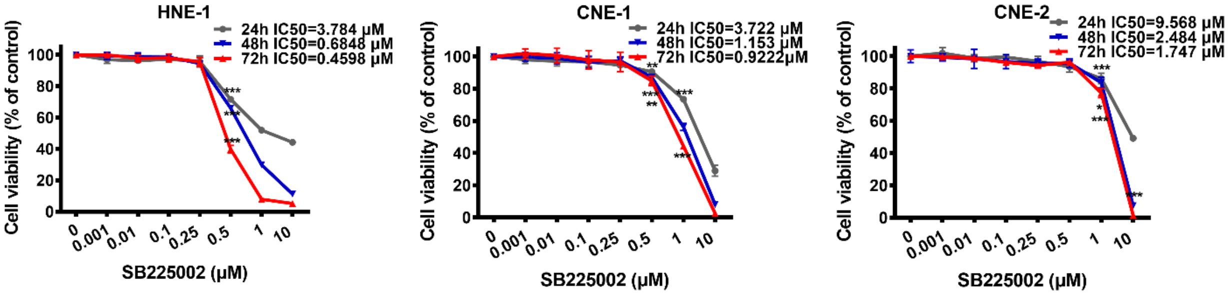

Supplementary Figure 1. SB225002 suppresses cell proliferation in vitro. SB225002 inhibited cell proliferation. HNE-1, CNE-1 and CNE-2 human nasopharyngeal cancer cells were treated with SB225002 for (24, 48 and 72 h), cell viability was evaluated by CCK8 assay (**p < 0.01; ***p < 0.001; Student's t-test). Values represent mean ± SD (n = 5).
