# Supplementary material for: Antitumor and Radiosensitization Effects of a CXCR2 Inhibitor in Nasopharyngeal Carcinoma
Source: Front Cell Dev Biol. 2021 May 26;9:689613. doi: 10.3389/fcell.2021.689613 (PMC8188356; doi:10.3389/fcell.2021.689613)
Supplement: Supplementary file 2 [file Image_2.pdf]

Supplementary Figure 2

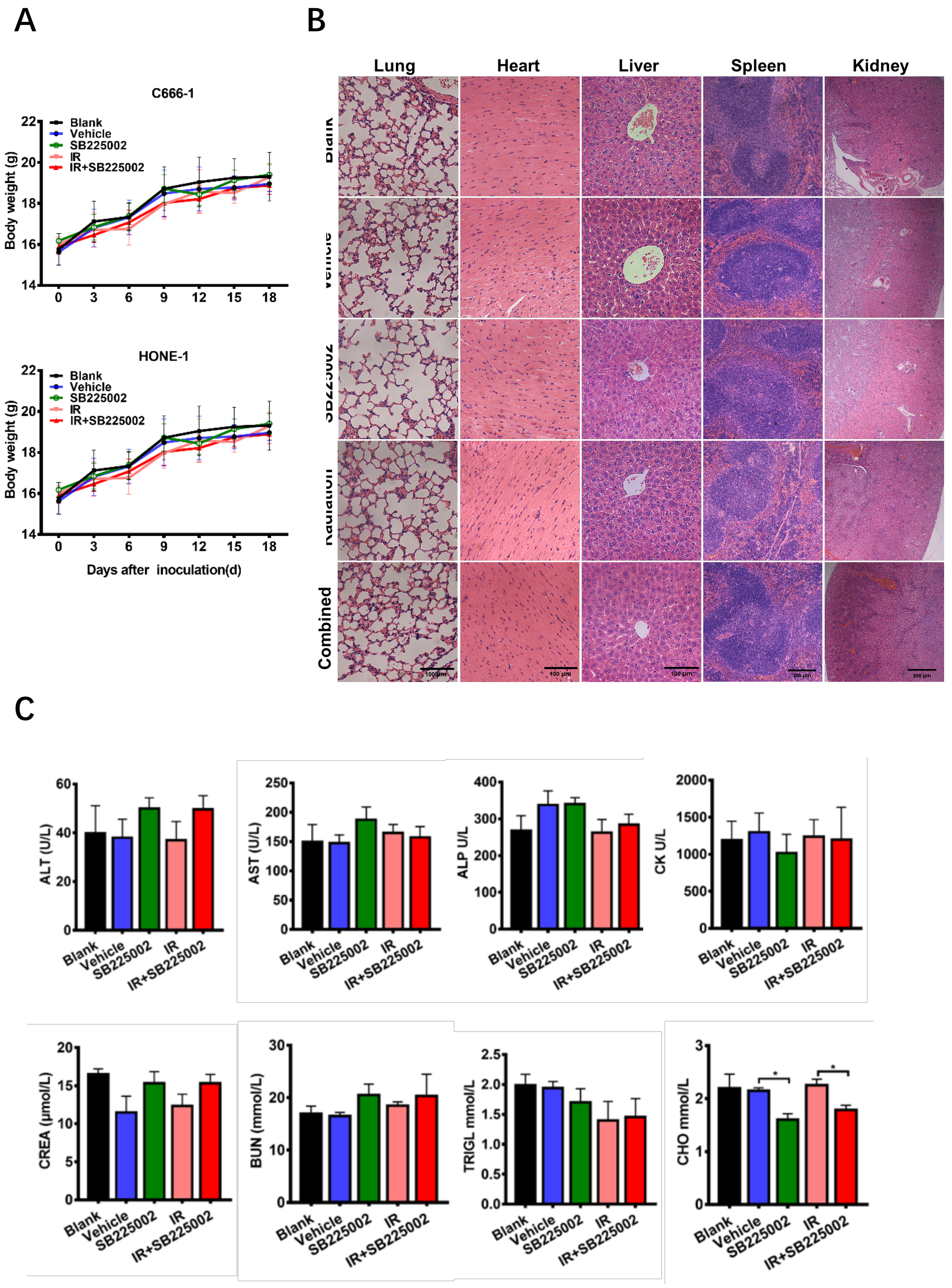

Supplementary Figure 2. Toxicity evaluation of treatment of SB225002 combined with radiotherapy. (A) The body weight changes in C666-1 and HONE-1 tumor-bearing nude

mice after SB225002 administrated. **(B)** H&E staining of mouse vital organs after treatment of SB225002 combined with radiotherapy in mice. Scale bar, 500 μm for kidney, 200 μm for spleen and 100 μm for other. **(C)** Biochemical detection of blood serum in C666-1 and HONE-1 tumor-bearing nude mice model. Values represent mean ± SD (n = 3; Student's t-test).
