# Supplementary material for: Antitumor and Radiosensitization Effects of a CXCR2 Inhibitor in Nasopharyngeal Carcinoma
Source: Front Cell Dev Biol. 2021 May 26;9:689613. doi: 10.3389/fcell.2021.689613 (PMC8188356; doi:10.3389/fcell.2021.689613)
Supplement: Supplementary file 3 [file Image_3.pdf]

Supplementary Figure 3

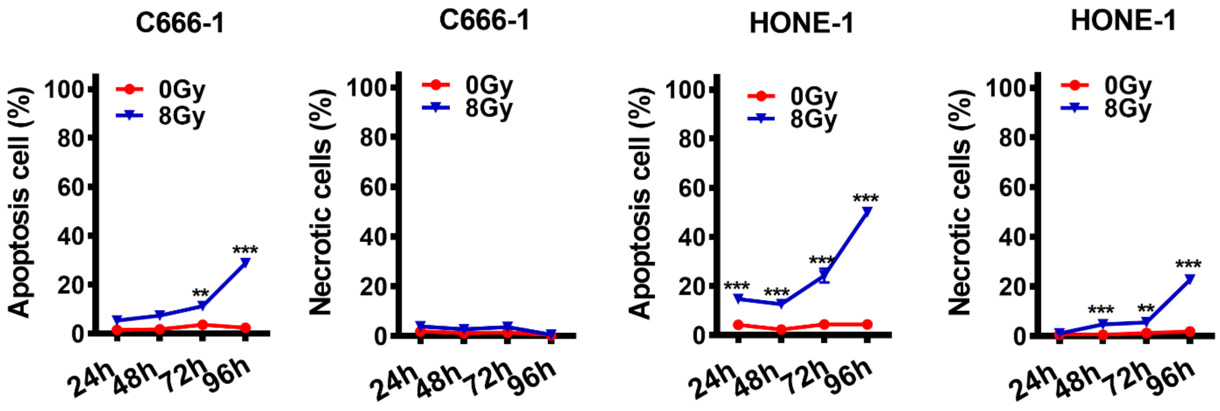

Supplementary Figure 3. The cell death induced by irradiation. C666-1 and HONE-1 cell was administrated with a 8Gy irradiation and the apoptosis and necrosis were examined using the AnnexinV-FITC/PI at indicated timepoints, as determined by flow cytometry.
