# Supplementary material for: Antitumor and Radiosensitization Effects of a CXCR2 Inhibitor in Nasopharyngeal Carcinoma
Source: Front Cell Dev Biol. 2021 May 26;9:689613. doi: 10.3389/fcell.2021.689613 (PMC8188356; doi:10.3389/fcell.2021.689613)
Supplement: Supplementary file 4 [file Table_1.docx]

**Table 1** Clinicopathological correlations of CXCR2 expressions in tumor cells of NPC patients determined by IHC

| characteristics | Number of Pts | Low expression | High expression | P value |
| --- | --- | --- | --- | --- |
| Age(years) | 99 | 47.97± 1.333 | 49.82 ± 2.498 | 0.2799 |
| ＜55 | 70 (69.7%) | 51 (71.8%) | 19 (67.9%) | 0.3275 |
| ≥55 | 29 (29.3%) | 20 (28.2%) | 9 (32.1%) |  |
| Gender |  |  |  |  |
| Male | 80 (79.8%) | 61 (85.9%) | 19 (67.9%) | 0.3767 |
| Female | 19 (19.2%) | 10 (14.1%) | 9 (32.1%) |  |
| TMN stage |  |  |  |  |
| Stage I-II | 55 (55.6%) | 41 (57.7%) | 14 (50.0%) | 0.5000 |
| Stage III-V | 44 (44.4%) | 30 (42.3%) | 14 (50.0%) |  |
| Lymph node metastasis |  |  |  |  |
| Yes | 70 (69.7%) | 50 (70.4%) | 20 (71.4%) | 0.2502 |
| No | 29 (29.3%) | 21 (29.6%) | 8 (28.6%) |  |
