# Supplementary material for: Antitumor and Radiosensitization Effects of a CXCR2 Inhibitor in Nasopharyngeal Carcinoma
Source: Front Cell Dev Biol. 2021 May 26;9:689613. doi: 10.3389/fcell.2021.689613 (PMC8188356; doi:10.3389/fcell.2021.689613)
Supplement: Supplementary file 5 [file Table_2.docx]

**Table 2** Clinicopathological correlations of CXCR2 expressions in stromal cells of NPC patients determined by IHC

| characteristics | Number of Pts | Low expression | High expression | P value |
| --- | --- | --- | --- | --- |
| Age(years) | 99 | 50.61 ± 1.667 | 45.96 ± 1.609 | 0.3903 |
| ＜55 | 70 (69.7%) | 34 (63.0%) | 36 (80.0%) | 0.1955 |
| ≥55 | 29 (29.3%) | 20 (37.0%) | 9 (20.0%) |  |
| Gender |  |  |  |  |
| Male | 80 (79.8%) | 47 (87.0%) | 33 (73.3%) | 0.1922 |
| Female | 19 (19.2%) | 7 (13.0%) | 12 (26.7%) |  |
| TMN stage |  |  |  |  |
| Stage I-II | 55 (55.6%) | 31 (57.4%) | 24 (53.3%) | 0.2716 |
| Stage III-V | 44 (44.4%) | 23 (42.6%) | 21 (46.7%) |  |
| Lymph node metastasis |  |  |  |  |
| Yes | 70 (69.7%) | 41 (75.9%) | 29 (64.4%) | 0.2233 |
| No | 29 (29.3%) | 13 (24.1%) | 16 (35.6%) |  |
