# Supplementary material for: Antitumor and Radiosensitization Effects of a CXCR2 Inhibitor in Nasopharyngeal Carcinoma
Source: Front Cell Dev Biol. 2021 May 26;9:689613. doi: 10.3389/fcell.2021.689613 (PMC8188356; doi:10.3389/fcell.2021.689613)
Supplement: Supplementary file 6 [file Table_3.docx]

**Table 3** Clinicopathological correlations of CXCL8 expressions in tumor cells of NPC patients determined by IHC

| characteristics | Number of Pts | Low expression | High expression | P value |
| --- | --- | --- | --- | --- |
| Age(years) | 99 | 50.73 ± 1.877 | 46.98 ± 1.508 | 0.8517 |
| ＜55 | 70 (69.7%) | 26 (65.0%) | 44 (74.6%) | 0.2502 |
| ≥55 | 29 (29.3%) | 14 (35.0%) | 15 (25.4%) |  |
| Gender |  |  |  |  |
| Male | 80 (79.8%) | 33 (82.5%) | 47 (79.7%) | 0.0933 |
| Female | 19 (19.2%) | 7 (17.5%) | 12 (20.3%) |  |
| TMN stage |  |  |  |  |
| Stage I-II | 55 (55.6%) | 23 (57.5%) | 32 (54.2%) | 0.0577 |
| Stage III-V | 44 (44.4%) | 17 (42.5%) | 27 (45.8%) |  |
| Lymph node metastasis |  |  |  |  |
| Yes | 70 (69.7%) | 24 (60.0%) | 46 (78.0%) | 0.3486 |
| No | 29 (29.3%) | 16 (40.0%) | 13 (22.0%) |  |
